# Controlling Ultrafast Excitations in Germanium: The Role of Pump-Pulse Parameters and Multi-Photon Resonances

**DOI:** 10.3390/ma19020408

**Published:** 2026-01-20

**Authors:** Amir Eskandari-asl, Adolfo Avella

**Affiliations:** 1 Dipartimento di Fisica “E.R. Caianiello”, Università degli Studi di Salerno, I-84084 Fisciano, SA, Italy; aeskandariasl@unisa.it; 2 CNR-SPIN, Unità di Salerno, I-84084 Fisciano, SA, Italy; 3 CNISM, Unità di Salerno, Università degli Studi di Salerno, I-84084 Fisciano, SA, Italy

**Keywords:** Dynamical Projective Operatorial Approach (DPOA), strong-field excitations, multi-photon absorption, residual excited-carrier population, residual absorbed energy

## Abstract

We employ the Dynamical Projective Operatorial Approach (DPOA) to investigate the ultrafast optical excitations of germanium under intense, ultrashort pump pulses. The method has very low resource demand relative to many other available approaches and enables detailed calculation of the residual electron and hole populations induced by the pump pulse. It provides direct access to the energy distribution of excited carriers and to the total energy transferred to the system. By decomposing the response into contributions from different multi-photon resonant processes, we systematically study the dependence of excited-carrier density and absorbed energy on key pump-pulse parameters: duration, amplitude, and photon energy. Our results reveal a complex interplay between these parameters, governed by resonant Rabi-like dynamics and competition between different multi-photon absorption channels. For the studied germanium setup, we find that two-photon processes are generally dominant, while one- and three-photon channels become significant under specific conditions of pump-pulse frequency, duration, and intensity. This comprehensive analysis offers practical insights for optimizing ultrafast optical control in semiconductors by targeting specific multi-photon pathways.

## 1. Introduction

In recent years, ultrafast pump–probe spectroscopy has emerged as a pivotal experimental advancement, enabling unprecedented access to the dynamics of condensed matter systems on femtosecond and even attosecond time scales [1,2,3,4,5,6,7,8]. By tracking the dynamics of photo-excited carriers, these experiments provide direct insights into the microscopic mechanisms governing electronic, spin, and lattice degrees of freedom far from equilibrium. From a technological perspective, such understanding is essential for the development of next-generation ultrafast optoelectronic and spintronic devices. Fundamentally, these methods allow for the real-time observation of symmetry breaking, coherence, and relaxation processes [9,10,11].

To theoretically address the complexity of ultrafast phenomena in realistic materials, we have recently developed the Dynamical Projective Operatorial Approach (DPOA) [7,12,13,14,15]. DPOA is an efficient operator-based formalism that enables the simulation of real-time evolution in multi-band systems under the application of pump fields. It provides direct access to key microscopic observables, such as the single-particle density matrix (SPDM) and band populations [16], inter-band coherences, and—in principle—every multi-time multi-particle response function, including time-resolved angle-resolved photoemission spectroscopy [12] and transient optical properties [13]. The formulation of DPOA is fully general, applicable to systems with arbitrary lattice structures, band numbers, and other complexities.

A central question in ultrafast optical excitations concerns the distribution of excited electrons and holes in energy and momentum space. Specifically: At which energies do the excited carriers reside? How many carriers are excited under a given pump pulse? And how much energy is transferred to the electronic system? Addressing these questions is crucial for advancing toward practical applications of ultrafast physics, such as optimizing pulse parameters for specific device functionalities. In this work, we apply DPOA to study the excitations in germanium driven by an intense ultrashort optical pulse, systematically analyzing the dependence of residual excited-carrier density and absorbed energy on pump-pulse parameters.

The manuscript is organized as follows. In Section 2, we present the theoretical framework, including the Hamiltonian in the dipole gauge, the construction of time-dependent hopping and dipole matrices via the Wannier basis, and the DPOA equations of motion. We also define the key quantities of interest: residual carrier populations, their photon-order decomposition, and the associated energy distributions. In Section 3, we report numerical results for germanium, examining the roles of pump-pulse duration, amplitude, and frequency in determining the excitation pathways and efficiency. Finally, in Section 4, we summarize our findings and discuss their implications for future studies and applications.

## 2. Theory

In this section, we present the relevant aspects of the DPOA framework for the analysis of residual excited-carrier populations and absorbed energy. Moreover, we define the fundamental quantities that enable us to conduct a comprehensive study of such excitations and achieve a deeper understanding of their underlying processes. It is worth noting that, in principle, this study is highly resource-intensive and could render many other approaches infeasible. In contrast, the extreme efficiency of the DPOA framework allows the required resources to remain well contained, thereby enabling analysis of a large portion of the relevant parameter space.

Working in the dipole gauge, the time-dependent Hamiltonian governing a pumped system is given by [12,13,17]

(1) $$H\left(t\right)=\sum_{k,n,n^{\prime }}c_{k,n}^{†}\left(t\right)\Xi_{k,n,n^{\prime }}\left(t\right)c_{k,n^{\prime }}\left(t\right),$$

where $c_{k,n}\left(t\right)$ annihilates an electron with crystal momentum k and band index *n* (including spin). The matrix $\Xi_{k}\left(t\right)$ originates from the first-quantized single-particle Hamiltonian and takes the form

(2) $$\Xi_{k}\left(t\right)=T_{k}\left(t\right)+eE_{\mathrm{pu}}\left(t\right)D_{k,\mathrm{pu}}\left(t\right),$$

with $D_{k,\mathrm{pu}}\left(t\right)=D_{k}\left(t\right)\cdot {\hat{u}}_{\mathrm{pu}}$. Here, $T_{k}\left(t\right)$ and $D_{k}\left(t\right)$ represent the time-dependent hopping and dipole matrices, respectively. The applied linearly polarized pump pulse is characterized by the vector potential

(3) $$A_{\mathrm{pu}}\left(t\right)=A_{\mathrm{pu}}\left(t\right){\hat{u}}_{\mathrm{pu}},$$

and the associated electric field

(4) $$E_{\mathrm{pu}}\left(t\right)=E_{\mathrm{pu}}\left(t\right){\hat{u}}_{\mathrm{pu}}=-\partial_{t}A_{\mathrm{pu}}\left(t\right){\hat{u}}_{\mathrm{pu}}.$$

The unit vector ${\hat{u}}_{\mathrm{pu}}$ specifies the pump-pulse polarization axis. The pump pulse is switched on from an initial time $t_{\mathrm{ini}}\to -\infty$, when the system resides in a thermal equilibrium state.

$T_{k}\left(t\right)$ and $D_{k}\left(t\right)$ are built by implementing the Peierls substitution in a localized Wannier basis and then rotated to the band basis that diagonalizes the equilibrium Hamiltonian. Their detailed expressions can be found in Refs. [12,13]. Operators expressed in the localized basis are hereafter marked with a tilde (˜). The rotation from the localized to the band basis is carried out by the unitary matrix $\Omega_{k}$. In compact notation, $\Omega_{k}^{†}\cdot {\tilde{T}}_{k}\cdot \Omega_{k}$ is diagonal, its entries being the band energies $\varepsilon_{k,n}$. For any matrix *M* that stands for either *T* or D, we have

(5) $$M_{k}\left(t\right)=\Omega_{k}^{†}\cdot {\tilde{M}}_{k+\frac{e}{\hbar }A_{\mathrm{pu}}\left(t\right)}\cdot \Omega_{k}.$$

The dot symbol · is used for both vector products in real space and matrix products in the electronic Hilbert space. In practical calculations on multi-band systems with dense k-meshes, it is numerically efficient to expand ${\tilde{M}}_{k+\frac{e}{\hbar }A_{\mathrm{pu}}\left(t\right)}$ as a power series in the vector potential through the Peierls expansion [12].

Within the DPOA formalism, the Heisenberg-picture annihilation operators $c_{k}\left(t\right)$ are related to their equilibrium values $c_{k}\left(t_{\mathrm{ini}}\right)$ via the projection matrices $P_{k}\left(t\right)$:

(6) $$c_{k}\left(t\right)=P_{k}\left(t\right)\cdot c_{k}\left(t_{\mathrm{ini}}\right).$$

Here, $c_{k}\left(t\right)$ is a column vector whose entries are the operators $c_{k,n}\left(t\right)$. The projection matrices satisfy the equation of motion [12]

(7) $$i\hbar \partial_{t}P_{k}\left(t\right)=\Xi_{k}\left(t\right)\cdot P_{k}\left(t\right),$$

which is solved numerically starting from the initial condition $P_{k}\left(t_{\mathrm{ini}}\right)=1$.

The electronic population in band *n* at momentum k and time *t* is given by

(8) $$N_{k,n}\left(t\right)=\langle c_{k,n}^{†}\left(t\right)c_{k,n}\left(t\right)\rangle .$$

Using the projection matrices, this can be expressed as

(9) $$N_{k,n}\left(t\right)=\sum_{n^{\prime }}P_{k,n,n^{\prime }}\left(t\right)f\left(\varepsilon_{k,n^{\prime }}\right)P_{k,n^{\prime },n}^{†}\left(t\right),$$

where $f\left(\varepsilon \right)={\left[e^{\beta \left(\varepsilon -\mu \right)}+1\right]}^{-1}$ is the Fermi distribution function, in which β is the inverse temperature and μ is the chemical potential.

We consider a Gaussian-enveloped pump pulse:

(10) $$A_{\mathrm{pu}}\left(t\right)=A_{0}e^{-\left(4\;\ln\;2\right)t^{2}/\tau_{\mathrm{pu}}^{2}}\sin\left(\omega_{\mathrm{pu}}t\right),$$

where $\tau_{\mathrm{pu}}$ is the full width at half maximum (FWHM) of the pulse (also referred to as the pump-pulse duration), and $\omega_{\mathrm{pu}}$ is the pump-pulse frequency. The quantity $\hbar \omega_{\mathrm{pu}}$ is dubbed the pump-pulse photon energy.

The residual electronic population after the pump pulse, $N_{k,n}^{\mathrm{res}}=N_{k,n}\left(t\to \infty \right)$, differs from the equilibrium value, $f(\varepsilon_{k,n})$, only for bands that are in an *l*-photon resonance (l=1,2,3,…) with the pump pulse. Here, t→∞ denotes times after the application of the pump pulse but before relaxation processes set in (tens of femtoseconds); such processes occur on time scales of hundreds of femtoseconds and are neglected in this work [7]. In particular, we can neglect the electron-phonon interaction (and, consequently, all indirect gaps), in contrast to many other cases that address longer timescales [9,18,19,20,21,22]. The residual excited-carrier population is

(11) $$\Delta N_{k,n}^{\mathrm{res}}=N_{k,n}^{\mathrm{res}}-f\left(\varepsilon_{k,n}\right).$$

Here, $\Delta N_{k,n}^{\mathrm{res}}>0$ corresponds to an electron in a conduction band (CB), while $\Delta N_{k,n}^{\mathrm{res}}<0$ corresponds to a hole in a valence band (VB). We consider a gapped semiconductor at low temperature, so that the VBs and CBs are well separated.

The residual excited-carrier population per unit cell, which can be referred to as the residual excited-carrier density, is

(12) $$\Delta N^{\mathrm{res}}=\frac{1}{M_{\mathrm{grid}}}\sum_{k,n_{C}}\Delta N_{k,n_{C}}^{\mathrm{res}},$$

where $n_{C}$ runs over all conduction bands and $M_{\mathrm{grid}}$ is the total number of k-points in the numerical grid.

The strength of an *l*-photon resonance for a given gap energy $\varepsilon_{\mathrm{gap}}$ is given by [12]

(13) $$\begin{array}{c} w_{l}\left(\varepsilon_{\mathrm{gap}}\right)=\exp\left[-\frac{\tau_{\mathrm{pu}}^{2}}{8\ln2\hbar^{2}l}{\left(\varepsilon_{\mathrm{gap}}-l\hbar \omega_{\mathrm{pu}}\right)}^{2}\right]. \end{array}$$

This expression resembles the squared amplitude of the $\varepsilon_{\mathrm{gap}}/\hbar$ component in the spectrum of the *l*-th power of the pump pulse, which is centered at $l\omega_{\mathrm{pu}}$.

The contribution of a specific VB, $n_{V}$, to the residual excited electron population in a given CB, $n_{C}$, via an *l*-photon process is estimated as [12]

(14) $$\Delta N_{k,n_{C},n_{V}}^{\mathrm{res}(l)}=\frac{\Delta N_{k,n_{V}}^{\mathrm{res}}w_{l}\left(\varepsilon_{k,n_{C}}-\varepsilon_{k,n_{V}}\right)}{\sum_{n_{V}^{\prime }}\Delta N_{k,n_{V}^{\prime }}^{\mathrm{res}}\sum_{l^{\prime }}w_{l^{\prime }}\left(\varepsilon_{k,n_{C}}-\varepsilon_{k,n_{V}^{\prime }}\right)}\Delta N_{k,n_{C}}^{\mathrm{res}}.$$

Summing over all VBs gives the total *l*-photon contribution to the residual excited-carrier population residing in $n_{C}$:

(15) $$\Delta N_{k,n_{C}}^{\mathrm{res}(l)}=\sum_{n_{V}}\Delta N_{k,n_{C},n_{V}}^{\mathrm{res}(l)}.$$

The residual excited-carrier density via *l*-photon resonant processes is

(16) $$\Delta N^{\mathrm{res}(l)}=\frac{1}{M_{\mathrm{grid}}}\sum_{k,n_{C}}\Delta N_{k,n_{C}}^{\mathrm{res}(l)}.$$

Analogous expressions hold for hole residual excited-carrier populations and densities, with the roles of CB and VB interchanged.

To resolve the energy distribution of the residual excited-carrier density, we define

(17) $$\rho^{\mathrm{res}}\left(\varepsilon \right)=\frac{1}{M_{\mathrm{grid}}}\sum_{k,n}\Delta N_{k,n}^{\mathrm{res}}L\left(\varepsilon -\varepsilon_{k,n}\right),$$

where $L\left(x\right)=\frac{1}{\pi }\frac{\lambda }{x^{2}+\lambda^{2}}$ is a Lorentzian with damping factor λ, chosen according to the k-grid density [13]. For an infinitely fine grid, $\lambda \to 0^{+}$ and hence, L(x)→δ(x). The *l*-photon resolved distribution is obtained by replacing $\Delta N_{k,n}^{\mathrm{res}}$ with $\Delta N_{k,n}^{\mathrm{res}(l)}$:

(18) $$\rho^{\mathrm{res}(l)}\left(\varepsilon \right)=\frac{1}{M_{\mathrm{grid}}}\sum_{k,n}\Delta N_{k,n}^{\mathrm{res}(l)}L\left(\varepsilon -\varepsilon_{k,n}\right).$$

Finally, the residual energy absorbed by the system per unit cell is

(19) $$E^{\mathrm{res}}=\frac{1}{M_{\mathrm{grid}}}\sum_{k,n}\Delta N_{k,n}^{\mathrm{res}}\varepsilon_{k,n},$$

which is independent of the energy origin due to particle conservation. The *l*-photon contribution is

(20) $$E^{\mathrm{res}(l)}=\frac{1}{M_{\mathrm{grid}}}\sum_{k,n}\Delta N_{k,n}^{\mathrm{res}(l)}\varepsilon_{k,n}.$$

## 3. Excitations in Germanium

**Figure 1:**
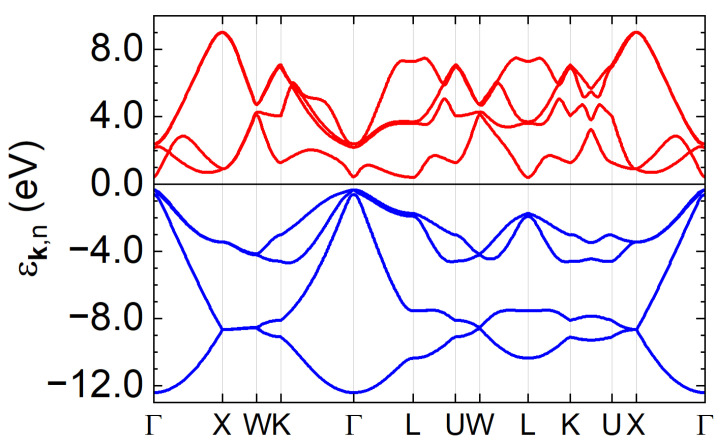
Equilibrium band structure of germanium along the main high-symmetry lines.

In this section, we present numerical results for optically pumped germanium, using the theoretical framework described in Section 2. The electronic structure is obtained from first-principles calculations performed with the Elk code [23]. The hopping parameters, ${\tilde{T}}_{k}$, and dipole matrix elements, ${\tilde{D}}_{k}$, are computed using Wannier90 [24], following the procedure detailed in Ref. [7]. We focus on a closed manifold of 16 $sp^{3}$ bands around the chemical potential the only one relevant to the excitations driven by the IR/visible pump pulse we considered), which captures the main band gap of approximately 800meV at the Γ point. The band structure, including spin-orbit coupling, is shown in Figure 1. The band splitting due to spin-orbit coupling is very small for the displayed bands. The chemical potential is set to zero energy.

The crystal orientation and pump-pulse polarization are chosen as in Ref. [7], with the field polarized along the [100] direction. The Brillouin zone is sampled on a 32×32×32 k-grid centered at Γ, and the Lorentzian broadening in Equation (17) is set to λ=0.079eV (equivalent to 0.12PHz). Unless otherwise stated, the default values of the pulse parameters are: $\tau_{\mathrm{pu}}=13.3\;fs$, $A_{0}=0.528\;V\;/\;nm\;fs$, and $\hbar \omega_{\mathrm{pu}}=1.55\;eV$. In all subsequent parameter scans, any parameter not being varied is held constant at its default value. Finally, the temperature is set to zero in our calculations: any physical finite temperature (around room temperature and reasonably above it) cannot change neither quantitatively nor qualitatively the results as the gap, which is about 800meV, amounts to a temperature of about 9300K.

**Figure 2:**
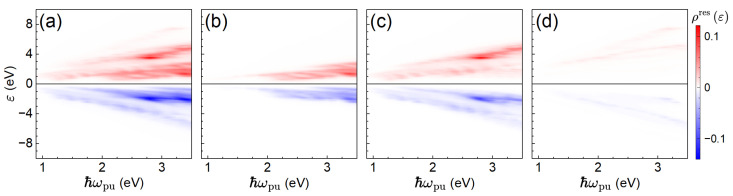
Energy distribution of residual excited-carrier density, $\rho^{\mathrm{res}}\left(\varepsilon \right)$, for different pump-pulse photon energies $\hbar \omega_{\mathrm{pu}}$. (**a**) Total distribution. (**b**–**d**) Contributions from one-, two-, and three-photon processes, respectively. Red (blue) color shows positive (negative) excess populations and corresponds to electron (hole) excitations. The vertical spacing between red and blue branches reflects the pump-pulse photon energy multiples, indicating resonant origins.

Figure 2a displays the total energy distribution of residual excited-carrier density, computed via Equation (17). The positive (red) and negative (blue) branches correspond to electron and hole excitations, respectively. Their separation in energy by multiples of $\hbar \omega_{\mathrm{pu}}$ clearly indicates that the excitations are due to *l*-photon resonant processes, where the resonance strength is governed by Equation (13). The distributions in Figure 2b–d decompose the signal into one-, two-, and three-photon contributions. Notably, for lower pump-pulse frequencies, two-photon processes dominate, consistent with earlier findings [7]. As the pump-pulse frequency increases, the role of one-photon excitations grows. Three-photon contributions remain relatively small; higher-order processes (l>3) are negligible and not shown here.

**Figure 3:**
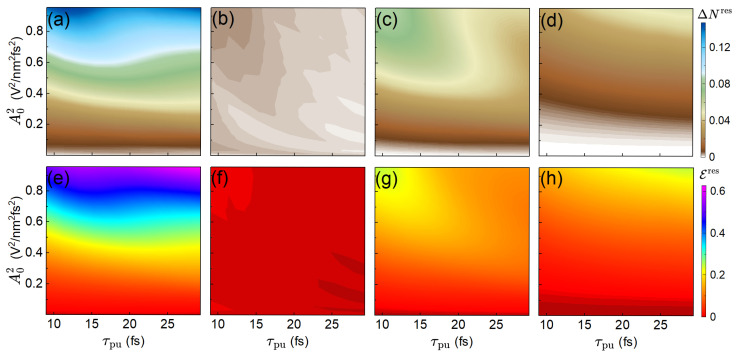
Residual excited-carrier density, $\Delta N^{\mathrm{res}}$, (upper row) and residual absorbed energy per unit cell, $E^{\mathrm{res}}$, (lower row) as functions of pump-pulse duration, $\tau_{\mathrm{pu}}$, and squared amplitude, $A_{0}^{2}$. (**a**,**e**) Total quantities. Contributions from (**b**,**f**) one-, (**c**,**g**) two-, and (**d**,**h**) three-photon processes, respectively.

Figure 3 explores the dependence of the residual excited-carrier density and absorbed energy per unit cell on pump-pulse duration and amplitude. Panels (a) and (e) show that, for fixed $\tau_{\mathrm{pu}}$, increasing $A_{0}$ generally enhances both $\Delta N^{\mathrm{res}}$ and $E^{\mathrm{res}}$, as expected for higher pump-pulse intensity. For small $A_{0}$, longer pulses monotonically increase the excited population and absorbed energy, whereas for larger amplitudes a non-monotonic Rabi-like behavior emerges: after an initial rise, the residual excited-carrier density decreases as $\tau_{\mathrm{pu}}$ increases. This reflects the increased Rabi frequency at higher amplitudes, which can drive populations back toward the ground state for sufficiently long pulses.

The photon-resolved panels reveal distinct behaviors. One-photon processes [panels (b, f)] are generally weak. Moreover, they are suppressed at longer $\tau_{\mathrm{pu}}$, especially at high amplitudes, where Rabi oscillations are strong. Two-photon processes [panels (c, g)] dominate across most parameter ranges and persist for longer pulse durations due to their smaller effective Rabi frequencies. Three-photon contributions [panels (d, h)] become significant only for high amplitudes and long pulses, where they can even exceed the two-photon signal. This sequential activation of higher-*l* processes with increasing $\tau_{\mathrm{pu}}$ stems from both differing Rabi frequencies and inter-channel competition: once higher-order resonances are activated, they reduce the available population for lower-order excitations. This is possible because at a single k-point we may have several different resonances simultaneously.

A noteworthy observation is that in the regime where three-photon processes dominate, their contribution in the absorbed energy per unit cell is substantially higher than the corresponding contribution of the two-photon resonances in their own regime, reflecting the larger number of energy quanta involved in multi-photon absorption.

**Figure 4:**
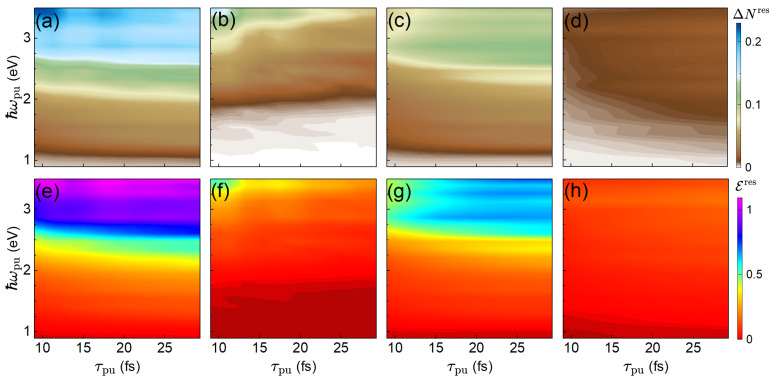
Residual excited-carrier density, $\Delta N^{\mathrm{res}}$, (upper row) and residual absorbed energy, $E^{\mathrm{res}}$, (lower row) as functions of pump-pulse duration, $\tau_{\mathrm{pu}}$, and pump-pulse photon energy, $\hbar \omega_{\mathrm{pu}}$. Columns as in Figure 3.

Figure 4 examines the joint dependence of the residual excited-carrier density and absorbed energy per unit cell on $\tau_{\mathrm{pu}}$ and $\hbar \omega_{\mathrm{pu}}$. The total excitations [panels (a, e)] generally increase with $\hbar \omega_{\mathrm{pu}}$, but exhibits pronounced dips and peaks that reflect the resonant matching between the pump-pulse frequency and specific band gaps across the Brillouin zone, as well as the variation of the strength of the coupling to the pump pulse. The detailed *l*-photon decompositions [panels (b–d, f–h)] show that each photon order has a distinct frequency dependence, governed by the available resonant transitions and their couplings to the pump pulse. The dependence on the pump-pulse duration again follows the pattern seen in Figure 3: shorter pulses favor lower-*l* processes, while longer pulses activate higher-*l* channels, moderated by Rabi decay and inter-process competition.

**Figure 5:**
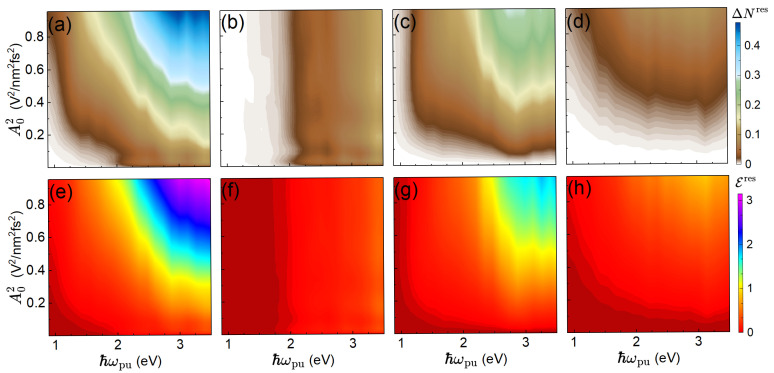
Residual excited-carrier density, $\Delta N^{\mathrm{res}}$, (upper row) and residual absorbed energy per unit cell, $E^{\mathrm{res}}$, (lower row) as functions of pump-pulse photon energy $\hbar \omega_{\mathrm{pu}}$ and squared amplitude $A_{0}^{2}$. Columns as in Figure 3.

Finally, Figure 5 shows how the excitations depend on both pump-pulse frequency and amplitude at fixed pulse duration. As in previous plots, increasing $A_{0}$ enhances excitations, while the frequency dependence reveals resonant structures that map out the relevant band gaps and coupling strength to the pump pulse. The photon-order resolved panels highlight how different absorption channels contribute across the parameter space. This detailed mapping provides practical guidance for selecting pump-pulse parameters to target specific excitation pathways in germanium or similar semiconductors.

## 4. Conclusions

In this work, we have presented a detailed theoretical and numerical investigation of residual carrier excitations in germanium driven by ultrafast optical pulses, using the Dynamical Projective Operatorial Approach (DPOA). Our study systematically addressed the fundamental questions of how many carriers are excited, at which energies they reside, and how much energy is absorbed by the electronic system as a function of pump-pulse parameters.

The energy distribution of residual excited-carrier density, $\rho^{\mathrm{res}}\left(\varepsilon \right)$, clearly exhibits a separation between electron and hole branches by multiples of the pump-pulse photon energy, confirming the dominance of *l*-photon resonant processes. The resonance strength, quantified by $w_{l}\left(\varepsilon_{\mathrm{gap}}\right)$, effectively captures the spectral selectivity of the Gaussian pump pulse.

The excitations show a strong dependence on pump-pulse duration ($\tau_{\mathrm{pu}}$) and amplitude ($A_{0}$). For high intensities, a non-monotonic, Rabi-like behavior emerges as a function of pulse duration, where excitations can be driven back toward the ground state for sufficiently long pulses. This highlights the importance of coherent light-matter interaction beyond simple perturbation theory.

Multi-photon decomposition reveals a distinct hierarchy and competition between absorption channels. In germanium, two-photon processes are generally the most efficient pathway across a wide parameter range. One-photon processes become significant at higher pump-pulse frequencies, while three-photon contributions require both high amplitude and long pulse duration to activate, underscoring the role of effective Rabi frequencies and state depletion.

The interplay between pump-pulse frequency and the electronic band structure leads to a rich, non-monotonic dependence of total residual excited-carrier density and absorbed energy on $\hbar \omega_{\mathrm{pu}}$. Peaks and dips correspond to optimal (or suboptimal) matching with specific band gaps and field coupling strength across the Brillouin zone.

The parameter-space maps of $\Delta N^{\mathrm{res}}$ and $E^{\mathrm{res}}$ provide a practical guide for tailoring ultrafast excitations. For applications requiring high carrier density with moderate energy input, the regime dominated by two-photon absorption is optimal. In contrast, targeting higher-energy excitations via three-photon processes requires carefully balancing pulse intensity and duration.

Our results explore the parameter-dependent landscape of ultrafast optical excitations in realistic multi-band materials. The insights gained here—particularly regarding the competition and activation conditions for different multi-photon orders—are not specific to germanium but represent general principles applicable to a wide class of semiconductors. This work establishes a foundation for the rational design of pump pulses to achieve desired electronic population distributions, a crucial step toward controlling ultrafast phenomena for next-generation optoelectronic and quantum devices. 

## Data Availability

The original data presented in the study are openly available in Zenodo at https://doi.org/10.5281/zenodo.18203588.
